# Combined Inositols, α-Lactalbumin, Gymnema Sylvestre and Zinc Improve the Lipid Metabolic Profile of Patients with Type 2 Diabetes Mellitus: A Randomized Clinical Trial

**DOI:** 10.3390/jcm12247650

**Published:** 2023-12-13

**Authors:** Alessandro Nani, Federico Bertuzzi, Elena Meneghini, Elena Mion, Basilio Pintaudi

**Affiliations:** 1 Department of Medical Biotechnology and Translational Medicine, University of Milan, 20133 Milan, Italy; 2 Department of Diabetology, Niguarda Hospital, 20162 Milan, Italy; 3 The Experts Group on Inositol in Basic and Clinical Research (EGOI), 00161 Rome, Italy

**Keywords:** myo-inositol, d-chiro-inositol, Gymnema sylvestre, α-lactalbumin, zinc, randomized clinical trial, type 2 diabetes mellitus, hypolipidemic effect, lipid metabolism

## Abstract

Type 2 diabetes mellitus (T2DM) is characterized by high blood glucose levels and lipid alterations. Besides pharmacological treatment, lifestyle modifications and nutraceuticals can be used to manage glucose and lipid profiles, which is crucial for preventing, or avoiding, serious consequences associated with the condition. This randomized controlled clinical trial on 75 patients with T2DM evaluated the effects of a combination of myo-inositol and d-chiro-inositol (40:1), α-lactalbumin, Gymnema sylvestre, and zinc on glucose and lipid profile. The intention-to-treat analysis displayed no significant differences in glucose parameters between the groups; however, the study group displayed reduced levels of total cholesterol (*p* = 0.01) and LDL (*p* = 0.03) after 3 months of supplementation. A subgroup analysis involving patients who did not modify their antidiabetic therapy, after 6 months displayed improved levels of total cholesterol (*p* = 0.03) and LDL (*p* = 0.04) in the study group versus placebo, along with a greater body weight reduction (*p* = 0.03) after 3 months. Furthermore, within the study group, levels of HDL (*p* = 0.03) and triglycerides (*p* = 0.04) improved after 3 months. These findings support supplementation with myo-inositol and d-chiro-inositol (40:1), α-lactalbumin, Gymnema sylvestre, and zinc as an adjuvant and safe strategy to manage the lipid profiles of patients with T2DM.

## 1. Introduction

Type 2 diabetes mellitus (T2DM) is the prevalent form of diabetes, representing approximately 90% of the overall cases worldwide [1]. Of concern, the International Diabetes Foundation estimates that the number of people living with T2DM will rise to 454 million by the year 2030 [2]. T2DM is a chronic metabolic disease characterized by high blood glucose levels due to impaired insulin secretion or functionality, especially *(i)* increased insulin resistance and/or *(ii)* deficient insulin secretion from β-pancreatic cells [3].

Of note, glucose and lipid metabolism are tightly correlated, and early clinical signs of T2DM include excess body weight and altered blood glucose levels. Indeed, insulin resistance may occur due to obesity, dysfunctional adipose tissue, chronic inflammation or a decrease in pancreatic β-cell mass. One of the major features of T2DM is diabetic dyslipidemia [4,5] (occurring in approximately 72–85% of patients) [6,7]. Such lipid aberration usually consists of increased levels of triglycerides, reduced levels of high-density lipoprotein (HDL) cholesterol, and the predominance of small, dense low-density lipoprotein (LDL) particles [8,9]. Considering that lipid anomalies play a central role in the genesis and progression of atherosclerosis, such an unbalanced lipid profile may lead to a substantially increased risk of cardiovascular disorders in comparison to subjects without T2DM.

First-line treatment of hyperglycemia in T2DM is typically managed by oral insulin-sensitizing drugs, such as metformin, but in the event of more serious cases (i.e., the failure of oral therapy, long-lasting hyperglycemia, or uncompensated T2DM) physicians may combine one or more glucose-lowering medications with different insulin regimens [10], in order to monitor and reduce the risk of complications (i.e., cardiovascular alterations, peripheral neuropathy and diabetic kidney disease) [11].

Besides pharmacological treatment, lifestyle modifications, such as a hypocaloric Mediterranean diet and physical activity, are strongly recommended. However, the adherence to lifestyle changes may be difficult and/or inefficacious; therefore, nutraceuticals provide important support to optimize the management of body weight, glycemic levels and lipid profile, and thus prevent dangerous T2DM complications [12].

Inositols, particularly myo-inositol (myo-Ins) and d-chiro-inositol (d-chiro-Ins), are natural molecules that positively modulate both glucose and lipid profiles through well-understood metabolic pathways [13]. Physiologically, myo-Ins acts as a second messenger in the cellular signaling of several hormones, including follicle-stimulating hormone (FSH), thyroid-stimulating hormone (TSH) and insulin [13]. Under insulin stimulation, myo-Ins may be converted into d-chiro-Ins by tissue-specific epimerases [14]. Moreover, both myo-Ins and d-chiro-Ins act as insulin-sensitizing agents by promoting glucose uptake and glycogen synthesis, respectively, thus reducing glycemia and consequently decreasing insulin requirements and plasma levels [15].

To date, several studies have demonstrated the insulin-sensitizing effect of inositols, specifically in their physiological 40:1 ratio, in various endocrine and metabolic disorders; however, to date, only one trial has evaluated the use of inositols in T2DM [13]. This pilot clinical trial reported that the administration of myo-Ins and d-chiro-Ins, in a 40:1 ratio, is an effective and safe strategy for boosting glycemic control in T2DM, significantly improving fasting blood glucose levels (control group: 192.6 ± 60.2 vs. study group: 160.9 ± 36.4; *p* = 0.02) and glycated hemoglobin (HbA1c) (control group: 8.6 ± 0.9 vs. study group: 7.7 ± 0.9; *p* = 0.02) after three months of supplementation [16]. The majority of studies involving inositols investigate their use in women affected by polycystic ovary syndrome (PCOS), an endocrine condition generally correlated with metabolic alterations [17]. The daily administration of inositols improves physiological glucose metabolism and reduces insulin resistance, in addition to improving lipid profile, including cholesterol and triglyceride levels, in overweight patients with PCOS [18,19,20]. A recent meta-analysis revealed that inositol supplementation induces a reduction in the body mass index (BMI) of overweight/obese subjects affected by PCOS (weighted mean difference = −0.41 kg/m^2^; 95% CI: −0.78, −0.04; *p* = 0.028) [21]. A further meta-analysis reported significantly reduced levels of triglycerides, total cholesterol and LDL cholesterol in patients with metabolic syndrome as a result of inositol treatment [22]. Such results draw attention to the use of these supplements as adjunct therapies for enhancing anthropometric indices, lipid profile and glycemic response [21].

Metabolic diseases like T2DM, obesity, and insulin resistance often correlate with intestinal inflammation and gut dysbiosis. Such conditions may consequently lead to lower absorption of micronutrients, like inositols, thus contributing to their depletion [1]. α-Lactalbumin (α-LA) is a whey protein that has been used to counteract “inositol resistance” resulting from poor gut bioavailability in a subset of patients by enhancing gut bioavailability of inositols [23,24,25]. Indeed, clinical trials confirmed that adding 50 mg of α-LA to a daily dosage of 4 g of inositols overcomes inositol resistance [26], increasing the efficacy of inositols in patients with PCOS [27]. Furthermore, α-LA may restore the physiological composition of the intestinal microbiota, thus preventing gut dysbiosis and the related metabolic consequences [28].

Gymnema sylvestre is commonly used in phytotherapy approaches to metabolic disease, and it is one of the most relevant botanicals for the treatment of diabetes and obesity [29]. Its components, notably, gymnemic acids, saponins and gurmarin, act directly to reduce glycemic levels, blood glucose levels, HbA1c and free fatty acids (FFAs), thus favoring normal insulin function [29,30,31]. In addition, preclinical studies and a clinical randomized trial have recently reported similar benefits with respect to body weight, BMI and very-low-density lipoprotein (VLDL) levels [31,32].

Among micronutrients, zinc exhibits a crucial role in influencing lipid and glucose metabolism by regulating insulin expression. In addition, plasma zinc levels are routinely reported to be low in diabetes patients [33]. Further evidence has supported the use of supplementation in addressing metabolic conditions, as zinc supplementation has been seen to reduce plasma levels of both LDL and triglycerides [34,35].

The administration of inositols, α-LA, Gymnema sylvestre and zinc may represent an adjunct therapy to routine therapeutic protocols in individuals with diabetes or other metabolic disorders. Therefore, this clinical trial aimed to evaluate the efficacy and safety of the aforementioned supplements in combination with typical hypoglycemic medication in adults with T2DM. Specifically, risk factors of a worsening prognosis, such as weight gain, cholesterol levels and obesity, were assessed by measuring plasma glucose and lipid markers.

## 2. Materials and Methods

### 2.1. Study Design and Participants

We conducted a monocentric, randomized, placebo-controlled, double-blind, two-arm, parallel-group clinical trial. The study was carried out in full accordance with the Guidelines for Good Clinical Practice and the Declaration of Helsinki, and it was registered with ClinicalTrials.gov (identifier: NCT04745780; approval number: 690-122019; board name: Comitato Etico Milano Area 3—ASST Grande Ospedale Metropolitano Niguarda). Prior to trial initiation, the protocol, the consent form and the patient information sheet were reviewed and approved according to local regulations by the appropriate health authority and by the institutional review board.

The study began in February 2021 and ended in December 2022 at the Outpatient Diabetes Center of the ASST Grande Ospedale Metropolitano (GOM) Niguarda, Milan (Italy).

All eligible participants were aged 18 years or older, with at least a one-year diagnosis of T2DM and HbA1c levels between 7.5 and 9.0% (58–75 mmol/mol), which were detected within the previous two months before initiation of the study. HbA1c values greater than 9.0% were excluded, as this indicated a strong glycemic alteration requiring multiple daily injections of insulin, while the women included in the study were menopausal for at least two years.

Key exclusion criteria included *(i)* ongoing multiple-dose insulin therapy; *(ii)* additional use/intolerance of nutraceuticals containing inositols, Gymnema sylvestre, α-LA and/or zinc; *(iii)* chronic exposure to systemic corticosteroids; *(iv)* known intestinal malabsorption; *(v)* previous major surgical treatments within six months before screening; and *(vi)* any other medical condition deemed prone to limit the candidate’s compliance with the trial.

All the patients provided written informed consent after receiving thorough informative leaflets.

The enrolled subjects were randomized into two groups according to a 1:1 ratio through a generator of random and unrepeated numbers that produced different α-numeric sequences with initials specific to both the study arms. Double masking was maintained throughout the trial, as neither fatal events nor major persistent disabilities occurred.

Of note, the enrollment process was heavily hindered by both eligible subjects’ poor availability and considerable modifications to outpatient care following the COVID-19 pandemic. Therefore, patient enrollment was terminated early, specifically after the 75th candidate. An ad hoc futility analysis was carried out through conditional power computation, which allowed the early termination of the trial owing to futility.

**Figure 1:**
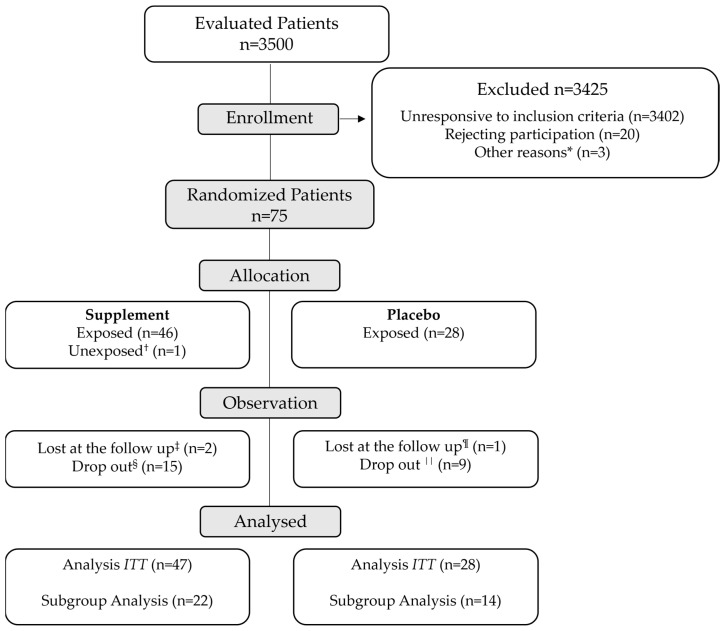
**Trial flow diagram.** * Patients excluded from the study due to chronic steroid therapy (n = 2) and known intestinal malabsorption (n = 1). ^†^ Patient treated with the supplement due to inconsistent indications from the general practitioner. ^‡^ Losses to follow-up due to absences at the following visits and inability to be contacted by phone. ^§^ Drop-outs due to family reasons (n = 5), low palatability (n = 4), diarrhea (n = 2), dysgeusia in COVID-19 (n = 1), hypersomnia (n = 1), persistent nausea (n = 1) and self-reported loss of therapeutic benefit (n = 1). ^¶^ Loss to follow-up due to a change in healthcare center (n = 1). ^||^ Drop-outs due to persistent headache (n = 1), onset of bladder cancer (n = 1), general malaise (n = 1), logistical problems (n = 1), polyuria (n = 1), bariatric surgery (n = 1), low therapeutic compliance (n = 1), recurrent angina decompensation (n = 1) and self-reported loss of therapeutic benefit (n = 1). The intention-to-treat (ITT) analysis refers to the entirety of the patient population, while the subgroup analysis refers to those patients who did not modify the antidiabetic therapy during the study and completed the trial.

No baseline washout nor home therapy consolidation over a lead-in period were contemplated; intervening treatment changes were consequently allowed throughout. Hypoglycemic rescue therapy was prescribed at the investigator’s discretion. The intervention group (n = 47) was treated with antidiabetic drugs and two sachets per day of Eudiamet^®^ 40:1 (Lo.Li. Pharma S.r.l., Rome, Italy), each containing myo-Ins (1950 mg), d-chiro-Ins (50 mg), α-LA (50 mg), Gymnema sylvestre (250 mg) and zinc (7.5 mg). Only one patient from this study branch was not prescribed the supplement on the advice of their general practitioner (Figure 1). In contrast, the control group (n = 28) received two placebo sachets a day. All sachets were consumed before lunch and dinner over a period of six months and were identical in appearance and flavor. According to current clinical practice, all the patients were recommended to observe an appropriate diet and practice regular physical activity.

The six-month follow-up was characterized by three timepoints: baseline (T0), after three months (T1) and six months (T2) from the randomization process. Individual socio-demographic, anthropometric and clinical information was collected at each timepoint. At T1 and T2, therapeutic adherence and possible adverse events were also investigated.

### 2.2. Study Outcomes

The primary outcome of the study was the evaluation of the degree of change in mean HbA1c levels from baseline to T1 and T2 in both study arms. Secondary efficacy outcomes included the following: (*i*) evaluation of blood levels of fasting glycemia and (*ii*) accurate monitoring and comparison of clinical and biochemical parameters (body weight, total cholesterol, HDL, LDL and triglycerides). Safety outcomes were systematically recorded from the consumption of the first dose of the supplement until the end of the follow-up period. An adverse event was defined as the onset or worsening of any undesirable symptom, sign or medical condition occurring after the beginning of the allocated treatment, regardless of cause. All adverse events were cataloged through the Medical Dictionary for Regulatory Activities (MedDRA^®^, version 26.0).

### 2.3. Statistical Analysis

The planned sample consisted of 128 patients, 64 per arm, and was calculated to reach a mean difference of 0.4 percentage points (4.4 mmol/mol) between the deltas of HbA1c in favor of the experimental group, also ensuring a significance level of 5% and a statistical power of 80%, together with a standard deviation (SD) of 0.8% (8.8 mmol/mol).

A first set of analyses was performed on the overall population, considering the initial treatment assignment, via the intention-to-treat (ITT) approach. In contrast, the safety analysis encompassed only those patients who were exposed to at least one dose of the dietary supplement.

Moreover, in light of the expected heterogeneity of our sample (arising from both different therapeutic regimens and a complex pathologic background), we prespecified further subgroup analyses. To mitigate the effect of varied background medications, other efficacy analyses comprised only those participants who needed no changes in their antidiabetic therapy throughout the study (Figure 1). Such patients also kept their antidiabetic therapy constant during a period spanning four to six months prior to the beginning of the study.

Statistical analysis was performed using GraphPad Software (version 8.0.1, La Jolla, CA, USA). The Shapiro–Wilk test was used to assess whether data were normally distributed or not. Intergroup comparisons were conducted either via *(i)* a Mann–Whitney U test or a *(ii)* a Student’s *t*-test, whilst *(iii)* the Wilcoxon test was used for intragroup comparisons at different timepoints. All descriptive parameters were stated as means ± SDs, as medians ± interquartile ranges [IQRs] or as percentages. A *p*-value of ≤0.05 was considered to be statistically significant.

## 3. Results

### 3.1. Patients, Follow-up and Futility Analysis

Patient enrollment was stopped prematurely after the 75th candidate, with the enrollment process being significantly impaired, partly by eligible subjects’ poor availability and partly by considerable modifications to outpatient care following the COVID-19 pandemic.

An ad hoc futility analysis was carried out through conditional power computation, which considered the intergroup observed mean decline in HbA1c (0.1 percentage points) with its SD (0.8%) and the prespecified type I error (α = 0.05). The result equaled 10%, a value far below the 80% power set up for sample size calculation. Furthermore, since the observed SD did not differ from the anticipated and prespecified SD, there was no need to resurvey our initial sample size. Hence, the conditional power quantified the primary outcome and allowed the early termination of the trial due to futility.

In the ITT analysis, a total of 75 participants were enrolled, including 47 individuals in the intervention group and 28 in the control group. No statistically significant differences in baseline characteristics were detected between the two groups (Table 1).

Additionally, among the patients with unmodified antidiabetic therapy, only 36 subjects completed the trial (22 in the experimental group versus 14 in the placebo group). As in the ITT population, baseline characteristics were not significantly different between the subgroups (Supplementary Table S2).

### 3.2. Glucose Metabolism

#### 3.2.1. ITT Analysis

Throughout the trial, the ITT analysis showed no statistically significant changes in glycemic parameters (HbA1c and fasting glycemia), neither intra- nor intergroup (Table 2).

#### 3.2.2. Subgroup Analysis

**Figure 2:**
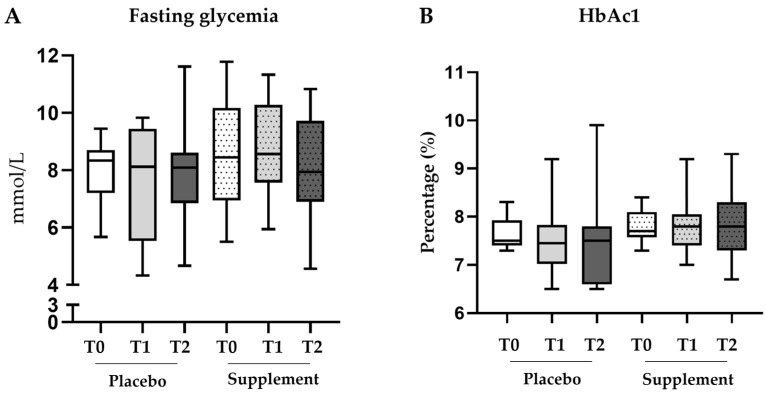
**Box plots of glycemic parameters in the subgroup analysis.** Fluctuations in the blood levels of fasting glycemia (**A**) and HbA1c (**B**) in the subgroup of patients with unmodified antidiabetic therapy (placebo n = 14; supplement n = 22). Statistical intergroup analysis was performed using the Mann–Whitney U test; statistical intragroup comparisons at different timepoints were performed using the Wilcoxon test.

The prespecified subgroup analysis, performed only on patients with an unmodified antidiabetic therapy, revealed no variations in blood levels of fasting glycemia (Figure 2A) or HbA1c (Figure 2B) at each of the trial timepoints. As reported in Table 3, medians were constant in both groups, thus confirming that the dietary supplement did not impair the glycemic parameters or the background antidiabetic therapy.

### 3.3. Lipid Metabolism and Body Weight

#### 3.3.1. ITT Analysis

**Figure 3:**
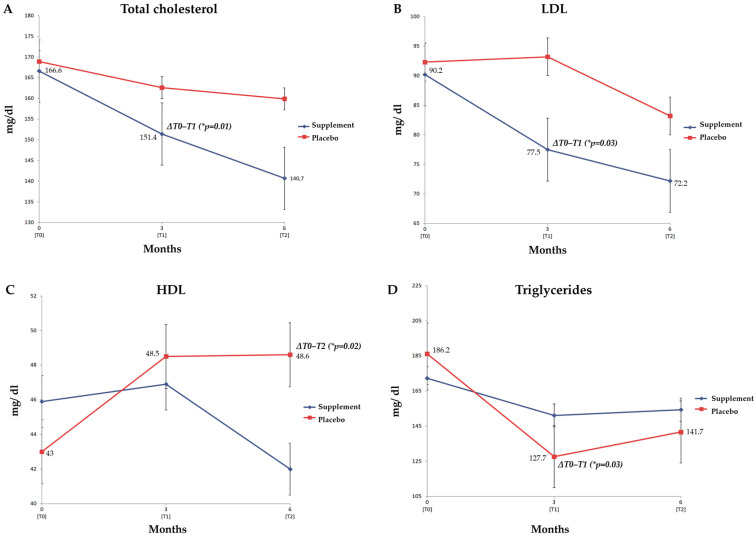
**Lipid parameters in the ITT population.** The four graphs represent the estimated mean with its standard error (SE) for each marker—total cholesterol (**A**), LDL (**B**), HDL (**C**) and triglycerides (**D**)—in both the arms throughout the follow-up period of six months. The analysis refers to all the enrolled patients (placebo n = 28; supplement n = 46), and the intragroup comparisons were performed via the Wilcoxon test (* *p*-value ≤ 0.05). Statistically significant intragroup deltas were foregrounded.

The ITT analysis indicated no statistically significant differences in lipid parameters (total cholesterol, HDL, LDL and triglycerides) between the two arms (Table 2). Both total cholesterol (Δ_T0–T1_ = −8.1 ± 24.4 mg/dL, *p* = 0.01) and LDL (Δ_T0–T1_ = −10.2 ± 26.5 mg/dL, *p* = 0.03) were significantly decreased in the intervention group during the first 3 months. In contrast, HDL significantly increased in the placebo group at the end of the study (Δ_T0–T2_ = 4.8 ± 4.5 mg/dL, *p* = 0.02), whereas triglycerides significantly dropped after three months (Δ_T0–T1_ = −20.7 ± 38.3 mg/dL, *p* = 0.03) (Figure 3).

#### 3.3.2. Subgroup Analysis

**Figure 4:**
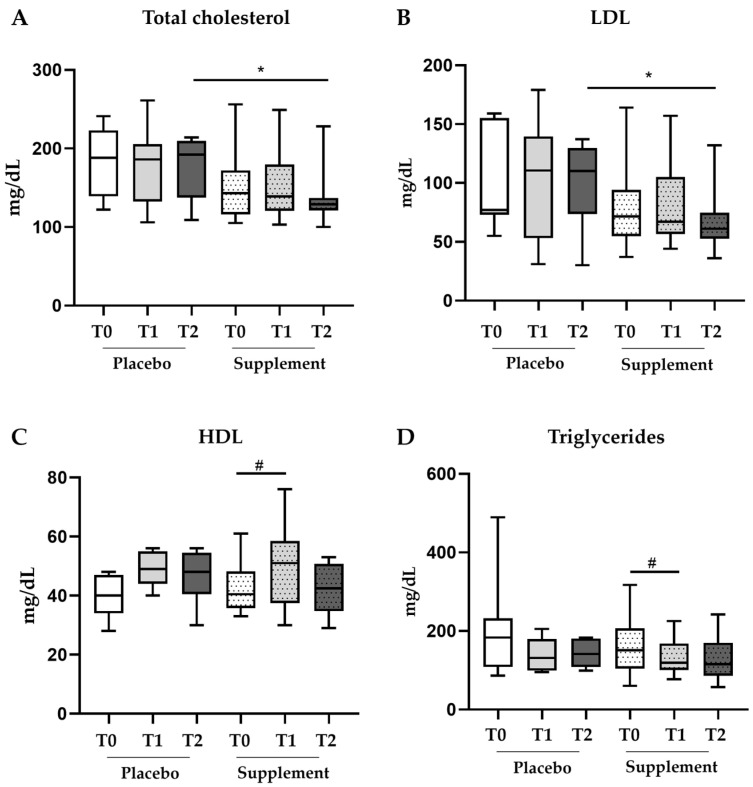
**Box plots of lipid parameters in the subgroup analysis**. Modifications in the blood levels of total cholesterol (**A**), LDL (**B**), HDL (**C**) and triglycerides (**D**) in the subgroup of patients with unmodified antidiabetic therapy (placebo n = 14; supplement n = 22). * *p* value ≤ 0.05 in the Mann–Whitney U test used for the intergroup analysis. # *p* value ≤ 0.05 in the Wilcoxon test used for the intragroup comparison.

Unlike the glycemic parameters, the prespecified subgroup analysis of blood lipid markers exhibited some improvements in the study group compared to the placebo. Specifically, at T2, the patients treated with the supplement experienced a significant reduction in blood levels of total cholesterol (*p* = 0.03) (Figure 4A). Indeed, as reported in Table 4, at the end of the follow-up, the median total cholesterol levels were 129.0 mg/dL [121.0–137.0 mg/dL] in the study group versus 192.0 mg/dL [137.5–209.5 mg/dL] with the placebo. Notably, both at baseline (T0) and after three months (T1), no significant difference existed between the groups (Figure 4A).

Likewise, at T2, blood levels of LDL were significantly lower in the study group (*p* = 0.04) (Figure 4B) with a median of 61.0 mg/dL [52.5–74.8 mg/dL] versus 110.0 mg/dL [73.50–129.5 mg/dL] in the placebo group (Table 4). However, blood levels of HDL were significantly higher only at T1 (versus T0) within the treatment group (*p* = 0.03) (Figure 4C). At this timepoint, levels of HDL rose from 40.5 mg/dL [35.8–48.3 mg/dL] at T0 to 51.0 mg/dL [37.5–58.5 mg/dL] at T1 (Table 4) and then went down to 42.5 mg/dL at T2.

Levels of triglycerides displayed a similar trend to HDL, showing significant improvement at only T1 (versus T0) within the treatment group (*p* = 0.04) (Figure 4D). In the treatment group, the median triglyceride levels were 151.0 mg/dL [104.0–206.5 mg/dL] at T0 versus 119.0 mg/dL [100.5–168.0 mg/dL] at T1 (Table 4). The triglyceride levels plateaued at T2 (115 mg/dL [86.0–169.5 mg/dL]) while remaining lower than the T2 value for the control group (141.0 mg/dL [108.0–180.5 mg/dL]), thus suggesting stability over the following months for this therapeutic target. No statistically significant changes in the serum levels of either HDL or triglycerides were observed in the placebo group.

**Figure 5:**
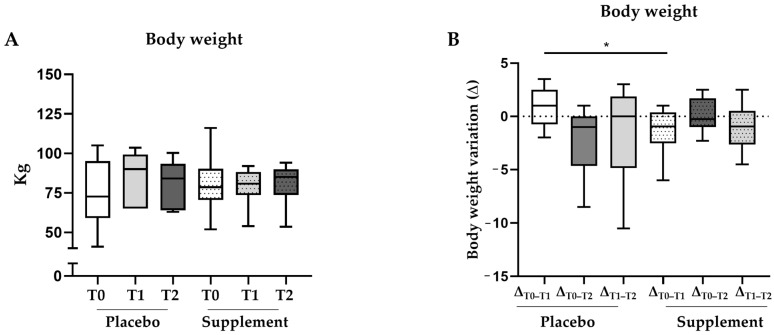
**Box plots of body weight and its deltas in the subgroup analysis at different timepoints.** Fluctuations in body weight (**A**) and its deltas (**B**) in the subgroup of patients with unmodified antidiabetic therapy (placebo n = 14; supplement n = 22). * *p*-value ≤ 0.05 in the Mann–Whitney U test used for the intergroup analysis.

In addition to the improvements in lipid metabolism, the six-month oral supplement regimen had a similarly positive impact on body weight. Even though no perceptible margin between the two subgroups in terms of overall weight of individuals within the cohort was observed (Figure 5A), the degree of change between the arms of the study was statistically significant after three months of treatment (*p* = 0.03) (Figure 5B). Specifically, the median Δ_T0–T1_ in the placebo group was 1.0 kg [−0.8–2.5 kg], while the median Δ_T0–T1_ in the study group was −0.95 kg [−2.5–0.4 kg] (Table 4), thus revealing a significant difference.

### 3.4. Safety Profile

No significant differences in terms of adverse events were recorded between the two arms (Supplementary Table S3). The most commonly experienced events were mild to moderate in nature and primarily consisted of gastrointestinal disorders (such as diarrhea or vomiting), without any substantial intergroup differences in occurrence (41.3% supplement vs. 35.7% placebo). Overall, serious adverse events were observed in only 3 of 46 patients within the supplement group and in 4 of 28 subjects treated with the placebo (6.5% vs. 14.3%); none of these events were fatal or linked to the trial nutraceuticals. No major hypoglycemic episodes were detected, aside from a serious nocturnal event promptly resolved by oral carbohydrate consumption. No amputations were required throughout the study, and no further clinically significant variations in other safety outcomes were documented. Of note, premature treatment discontinuation due to adverse events occurred more frequently in the control group (17.9% vs. 10.9%).

## 4. Discussion

Our study represents the first randomized clinical trial highlighting that a dietary supplement consisting of inositols, α-LA, Gymnema sylvestre and zinc may be a safe and effective strategy for improving the lipid profiles of subjects with T2DM. Even though the IIT analysis displayed improvements within the study group, a subgroup analysis revealed improved levels of lipid markers in the study group compared with the placebo. Moreover, the supplementation group demonstrated greater weight loss compared with the placebo.

T2DM is a chronic metabolic disorder characterized by a condition of hyperglycemia that arises from impaired insulin secretion and/or from peripheral resistance to insulin action. Long-lasting hyperglycemia, in addition to increased levels of triglycerides and low levels of HDL, may have serious consequences for the function of various organs and systems, thus increasing up to four-fold the risk of cardiovascular diseases [36]. Glucose and lipid metabolism are strictly correlated [37]: the accumulation of fat, especially visceral adiposity, is considered among the major contributors to insulin resistance, and, likewise, insulin resistance is a major contributor to dyslipidemia, creating a vicious circle [38].

On this premise, one of the objectives of the clinical management of T2DM is providing optimal glycemic control in addition to reversing lipid alterations that are integral hallmarks of diabetes. Physicians often recommend lifestyle programs, such as a hypocaloric Mediterranean diet or physical activity, but, unfortunately, the adherence to lifestyle changes may be difficult and inefficacious. As a result, additional nutraceutical interventions may be necessary to support pharmacological therapies and optimize their therapeutic effect.

Among several natural compounds with beneficial effects on glucose and lipid metabolism, inositols, α-LA, Gymnema sylvestre and zinc have a prominent role due to their positive effects on glucose metabolism, insulinemia, intestinal inflammation and lipid profile. Despite difficulties in the enrollment, this clinical study demonstrated for the first time that the oral administration of myo-Ins and d-chiro-Ins (40:1), α-LA, Gymnema sylvestre, and zinc improves lipid metabolic markers in patients with T2DM who are poorly responsive to hypoglycemic treatments.

Our results agree with the findings of a recent clinical study from Basciani and colleagues, in which the authors demonstrated that the adjuvant use of such a combination improved the lipid profiles of patients with metabolic syndrome [39]. In particular, a hypocaloric Mediterranean diet with supplementation of myo-Ins and d-chiro-Ins (in their physiological ratio 40:1), α-LA, Gymnema sylvestre, and zinc is a successful combination to restore a healthy lipid profile compared to diet alone.

Inositols indeed play a key role in the management of metabolic disorders, as evidenced by the literature, which has demonstrated the safety of the dosage of 4 g of inositols in their 40:1 ratio in dysmetabolic patients, highlighting their insulin-sensitizing effect [2,3,4]. Even though most studies involving inositols concern women affected by PCOS, they highlight an effect on glucose metabolism, insulin resistance and lipid profile [13]. A recent review investigated and clarified that in conditions of hyperglycemia, higher levels of glucose may competitively inhibit the absorption of myo-Ins, leading to an increase in myo-Ins degradation and urinary excretion, thus causing a potential deficiency in patients with diabetes [40]. Other studies corroborated their effects on lipid profiles, including an improvement in cholesterol and triglyceride levels, in overweight patients with PCOS [19,20]. Additionally, a recent meta-analysis focused also on the effects of inositols on body weight and lipid profiles [21,22]. A recent in vitro study indicated a mechanism of action of inositols on adipose tissue, thus corroborating the scientific interest in such molecules for their antiobesity activity [41]. Myo-Ins and d-chiro-Ins may induce the trans-differentiation of white adipose tissue (WAT) to brown adipose tissue (BAT), usually altered in the event of obesity. Specifically, the authors also correlated such induced increased trans-differentiation with an increased expression of molecular markers, specifically: *(i)* peroxisome proliferator-activated receptor gamma (PPAR-ɤ) that favors the transition from white to brown adipocytes and which is highly expressed in BAT [42,43]; *(ii)* uncoupling protein 1 (UCP-1), the primary BAT marker; *(iii)* and the mitochondrial copy number, in addition to the oxygen consumption ratio. Such results pave the way for a possible mechanism that explains the therapeutic effects of inositols in reducing body weight and improving metabolic complications. By highlighting the action of inositols in inducing the browning of both subcutaneous and visceral adipocytes, they also confirmed trans-differentiation from BAT to WAT as one of the main targets for further therapies against obesity and metabolic diseases. Given the inherent link between obesity and T2DM, this may play a role in reducing the risk of further progression of T2DM and requires further exploration.

As reported in previous studies, the use of α-LA in combination with inositols improves their intestinal absorption and therapeutical effectiveness. Furthermore, by passing unchanged through the stomach, α-LA may exert protective effects against intestinal inflammation and insulin resistance by stimulating beneficial bacterial strains [42]. A preclinical study by Boscaini et al. [28] demonstrated that a diet enriched in α-LA stimulated beneficial strains, such as *Lactobacillus acidophilus*, *Bifidobacterium short*, *Bifidobacterium longum* and *Bifidobacterium infantis*, whose abundance correlated with significant improvements in HbA1c and HOMA indexes in patients with T2DM, suggesting the positive effects of α-LA in restoring altered metabolic parameters [44]. Considering that dysmetabolic conditions such as obesity and insulin resistance may correlate with intestinal inflammation and gut dysbiosis, this stimulation of beneficial microorganisms is crucial for optimizing the therapeutic effects of inositols in T2DM.

There are a wide range of studies on the positive effects of Gymnema sylvestre on glucose alterations. Some authors have speculated upon the mechanism of action, indicating that gymnemic acids may stimulate the pancreas to produce insulin, in addition to promoting the regeneration of β-pancreatic cells [31]. Several studies also observed that gymnemic acids may reduce the intestinal absorption of glucose, restoring the physiological balance of glucose and insulin [29,43,45]. Regarding the lipid profile, a preclinical study conducted in rats with hyperlipidemia orally treated with Gymnema sylvestre demonstrated reduced total cholesterol, LDL and triglycerides [46]. Considering the correlation between obesity and T2DM, Gymnema’s activity on lipid metabolism may offer a cost-effective additional therapy for the treatment of T2DM [47].

Finally, several authors have reported zinc deficiency as an important risk factor in the development of T2DM; indeed, diabetic patients exhibit decreased concentrations of zinc compared to healthy people [48]. Overall zinc deficiency correlates with the worsening of related metabolic disturbances, such as insulin resistance, inflammation and altered lipid profile [49,50]. Previous studies indicated that zinc may induce the inhibition of proinflammatory cytokine expression, playing a role in ROS neutralization as well as in glucose and lipid metabolism. Its supplementation resulted in a significant decrease in body weight and BMI values as well as serum triglyceride concentrations [51], thus paving the way for zinc supplementation in the treatment of metabolic disorders.

While the supplement described in the study did not reduce serum levels of glycemia and HbAc1, it demonstrated the safety of using such molecules in patients with T2DM; furthermore, it did not interfere with background diabetic parameters, nor did it impair glycemic parameters. The significant reductions within the treated group in terms of body weight, cholesterol and triglycerides could represent a meaningful aid to patients for whom adopting a diet and exercise regimen may not be sufficient. The change in body weight is of particular interest due to inositols’ role in WAT/BAT differentiation, representing a source of inspiration for further investigations into how supplementation of the above molecules affects other obesity and metabolic markers.

It should be noted that the limited number of evaluated patients in the subgroup analysis and the difficulties in continuing clinical activity due to COVID-19 represent limitations of the study. In addition, the early cessation of the study owing to futility caused a non-intentional imbalance in the numbers of randomized individuals between the two arms. This was not a result of incorrect randomization procedures nor other randomization factors, but of the circumstances the COVID-19 pandemic presented. It is notable that the population represented in the study was of advanced age; therefore, it may not be entirely representative of general diabetic populations. Therefore, other additional studies will be useful to corroborate the reported evidence and to strengthen the use of such natural molecules in the management of patients with T2DM.

## 5. Conclusions

This randomized clinical trial highlighted for the first time the benefits of the reported combined natural molecules on the lipid profiles of patients with T2DM. In the treatment of T2DM, it is vitally important to not only control hyperglycemia, but also to monitor the lipid profile to prevent or avoid additional health complications. The findings of this study may support the use of dietary supplements based on myo-Ins and d-chiro-Ins (in their physiological ratio 40:1), α-LA, Gymnema sylvestre, and zinc as an adjuvant therapy for improving lipid disorders in patients with T2DM already undergoing hypoglycemic treatments.

## Figures and Tables

**Table 1:** **Baseline characteristics of the ITT population.** Data are provided for all the enrolled patients and are reported as n (%) or medians [IQRs], unless otherwise specified. The statistical analysis was performed through the Mann–Whitney U test. Other measured baseline characteristics are described within the supplementary data in Table S1.

Parameter	Supplement (n = 47)	Placebo (n = 28)	*p*-Value
Age (years)	67 [62–74]	68.5 [62.3–76]	0.9
Gender (%)			
Male	70.2	71.4	
Female	29.8	28.6	
Ethnicity (%)			
Caucasian	89.4	92.8	
Other	10.6	7.1	
Body weight (kg)	77.7 [70–90]	89.5 [70.5–96.8]	0.2
BMI (kg/m^2^)	28 [24.2–30.6]	29.3 [25.2–32.9]	0.2
BMI classes (%)			0.45
<25 kg/m^2^	27.6	21.4	
≥25 ∧ <30 kg/m^2^	40.4	32.1	
≥30 kg/m^2^	31.9	46.4	
Fasting glycemia (mmol/L)	9.3 [7.3–10.2]	8.6 [7.7–9.4]	0.3
HbA1c (%)	7.7 [7.5–8.1]	7.6 [7.4–8.3]	0.7
Total cholesterol (mg/dL)	160 [128.5–201.5]	170.5 [139.3–188.8]	0.8
HDL (mg/dL)	44 [35–56]	41.5 [33.3–49.5]	0.4
LDL (mg/dL)	84 [57–122]	90.5 [68.5–111]	0.7
Triglycerides (mg/dL)	154 [98–206]	156 [125.5–208.5]	0.8

∧ means BMI ranging from 25 and 30 kg/m^2^.

**Table 2:** **Efficacy outcomes in the ITT population.** Data are derived from all the enrolled patients and are reported as medians [IQRs], having been analyzed using the Mann–Whitney test, unless otherwise specified.

Parameter	3 Months (T1)				6 Months (T2)	
	Supplement (n = 46)	Placebo (n = 28)	*p*-Value	Supplement (n = 46)	Placebo (n = 28)	*p*-Value
Body weight (kg)	77.5 [72–85]	87 [71.5–94.3]	0.10	77.5 [72.9–87.9]	84.5 [73.8–90]	0.49
Fasting glycemia (mmol/L)	8.6 [7.5–10.1]	8.8 [7–9.6]	0.7	8.6 [7.1–9.7]	8.3 [7.2–10]	0.99
HbA1c (%)	7.8 [7.1–8]	7.7 [7–8.2]	0.66	7.8 [7.4–8.2]	7.5 [6.6–8]	0.15
Total cholesterol (mg/dL)	140 [119.8–165.8]	149 [120.5–203.5]	0.48	133 [111.5–168.5]	154 [119–200]	0.11
HDL (mg/dL)	48.5 [33.8–57.5]	48.5 [40.8–56]	0.54	40 [33.8–48.5]	49 [41–54]	0.09
LDL (mg/dL)	67.5 [55–89.3]	83 [51.5–129.5]	0.33	64 [45.5–92]	87 [54–118]	0.26
Triglycerides (mg/dL)	124 [101–174]	108.5 [92–169]	0.5	129 [98–171.3]	127 [102.3–181.3]	0.97

**Table 3:** **Glycemic parameters in the subgroup analysis.** Data are reported as medians [IQRs], unless otherwise specified. All timepoints (T0 = baseline; T1 = 3 months; T2 = 6 months) for both subgroups are displayed.

Parameter	Supplement (n = 22)				Placebo (n = 14)	
	T0	T1	T2	T0	T1	T2
Fasting glycemia (mmol/L)	8.4 [6.9–10.2]	8.6 [7.6–10.3]	7.9 [6.9–9.8]	8.3 [7.2–8.7]	8.1 [5.5–9.4]	8.1 [6.8–8.6]
HbA1c (%)	7.7 [7.6–8.1]	7.8 [7.4–8.1]	7.8 [7.3–8.3]	7.5 [7.4–7.9]	7.45 [7.0–7.8]	7.5 [6.6–7.8]

**Table 4:** **Lipid parameters and body weight in the subgroup analysis**. Data are reported as medians [IQRs], unless otherwise specified. All the timepoints (T0 = baseline; T1 = 3 months; T2 = 6 months) for both subgroups are displayed.

Parameter	Supplement (n = 22)				Placebo (n = 14)	
	T0	T1	T2	T0	T1	T2
Total cholesterol (mg/dL)	143 [116–172]	139 [120.5–179.5]	129 [121–137]	188 [139–223]	186 [132.5–205.5]	192 [137.5–209.5]
LDL (mg/dL)	71.5 [54.8–94.8]	67 [56.5–105]	61 [52.5–74.8]	77 [73–155]	110.5 [53–139.5]	110 [73.5–129.5]
HDL (mg/dL)	40.5 [35.8–48.3]	51 [37.5–58.5]	42.5 [34.8–50.8]	40 [34–47]	49 [44–55]	48 [40.5–54.5]
Triglycerides (mg/dL)	151 [104–206.5]	119 [100.5–168]	115 [86–169.5]	183 [108–232]	131 [99–179]	141 [108–180.5]
Body weight (kg)	78 [73.5–90]	76.5 [73–86.5]	80 [70.6–88.3]	87 [64.1–99]	90 [69–96.5]	83.5 [71–96.5]
Body weight variation (Δ)	Δ_T0–T1_ −0.95 [−2.5–0.4]	Δ_T0–T2_ −0.3 [−1–1.7]	Δ_T1–T2_ −0.95 [−2.6–0.5]	Δ_T0–T1_ 1 [−0.8–2.5]	Δ_T0–T2_ −1 [−4.7–0]	Δ_T1–T2_ 0 [−4.9–1.9]

## Data Availability

Data are available from the corresponding author on reasonable request.

## Supplementary Materials

The following supporting information can be downloaded at: https://www.mdpi.com/article/10.3390/jcm12247650/s1, Table S1: Additional baseline characteristics in the ITT population. Table S2: Main baseline characteristics only in those patients with an unmodified antidiabetic therapy. Table S3: Overview of the adverse events occurring during the entire study.
