# Supplementary material for: Combined Inositols, α-Lactalbumin, Gymnema Sylvestre and Zinc Improve the Lipid Metabolic Profile of Patients with Type 2 Diabetes Mellitus: A Randomized Clinical Trial
Source: J Clin Med. 2023 Dec 13;12(24):7650. doi: 10.3390/jcm12247650 (PMC10743679; doi:10.3390/jcm12247650)
Supplement: Supplementary file 1 [file jcm-12-07650-s001.zip › Supplementary Table S1.pdf]

| Parameter | | Supplement (n=47) | Placebo (n=28) | p value |
|---|---|---|---|---|
| Smoke (%) | | | | 0.13 |
| | Never | 21.3 | 35.7 | |
| | Past | 55.3 | 57.1 | |
| | Actual | 23.4 | 7.1 | |
| Familiarity for diabetes (%) | | 72.3 | 64.3 | 0.60 |
| Arterial hypertension (%) | | 74.5 | 78.6 | 0.78 |
| Ischemic heart disease (%) | | 27.6 | 35.7 | 0.61 |
| Coronary artery disease (%) | | 38.3 | 35.7 | 0.99 |
| Carotid stenosis (%) | | 23.4 | 25.0 | 0.99 |
| Peripheral chronic obliterating arterial disease (%) | | 6.4 | 3.4 | 0.99 |
| Cerebral vascular disease (%) | | 10.6 | 3.4 | 0.40 |
| Thrombosis (%) | | 21.3 | 25.0 | 0.78 |
| Chronic kidney disease (%) | | 27.6 | 32.1 | 0.79 |
| Diabetic retinopathy (%) | | 12.8 | 17.8 | 0.74 |
| Diabetic neuropathy (%) | | 19.1 | 14.3 | 0.76 |
| Neoplasia (%) | | 14.9 | 14.3 | 0.99 |
| Chronic liver disease (%) | | 8.5 | 10.7 | 0.99 |
| Hepatosteatosis (%) | | 27.6 | 17.8 | 0.41 |
| Chronic pulmonary disease (%) | | 4.2 | 14.3 | 0.19 |
| Hypoglycemic drugs (%) | | | | |
| | Basal insulin | 27.6 | 35.7 | 0.60 |
| | Metformin | 83.0 | 75.0 | 0.55 |
| | Pioglitazone | 4.2 | 0.0 | 0.53 |
| | Repaglinide | 2.1 | 3.4 | 0.99 |
| | Sulphonylureas | 14.9 | 14.3 | 0.99 |
| | Acarbose | 12.8 | 14.3 | 0.99 |
| | SGLT2 inhibitors | 46.8 | 57.1 | 0.48 |
| | DPP-4 inhibitors | 34.0 | 21.4 | 0.30 |
| | GLP-1 agonist receptors | 23.4 | 35.7 | 0.29 |
| Cardiovascular therapies (%) | | | | |
| | Anti-hypertensive | 72.3 | 78.6 | 0.60 |

| | | | |
|---|---|---|---|
| ACE-inhibitors | 27.6 | 39.3 | 0.32 |
| Sartans | 38.3 | 25.0 | 0.31 |
| B-blockers | 53.2 | 53.6 | 0.99 |
| Calcium-antagonist | 36.2 | 28.6 | 0.61 |
| Diuretics | 31.9 | 32.1 | 0.99 |
| Apha1-blockers | 8.5 | 21.4 | 0.16 |
| Statins | 65.9 | 67.8 | 0.40 |
| Ezetimibe | 8.5 | 10.7 | 0.40 |
| Omega | 17.0 | 17.8 | 0.42 |
| Fibrates | 4.3 | 3.4 | 0.42 |
| Antiplatelet agents | 51.1 | 42.8 | 0.63 |
| Anticoagulants | 6.4 | 0.0 | 0.29 |

**Table S1. Additional baseline characteristics in the ITT population.** Other measured baseline characteristics in addition to those in Table 1. Data refers to all the enrolled patients, they are reported as n (%), unless otherwise specified. Statistical test used was the non parametric Mann-Whitney U test.
