# Supplementary material for: Combined Inositols, α-Lactalbumin, Gymnema Sylvestre and Zinc Improve the Lipid Metabolic Profile of Patients with Type 2 Diabetes Mellitus: A Randomized Clinical Trial
Source: J Clin Med. 2023 Dec 13;12(24):7650. doi: 10.3390/jcm12247650 (PMC10743679; doi:10.3390/jcm12247650)
Supplement: Supplementary file 1 [file jcm-12-07650-s001.zip › Supplementary Table S2.pdf]

Parameter	Supplement (n=22)	Placebo (n=14)	p value
Body weight (kg)	78 [73.5 - 90]	87 [64.1 - 99]	0.35
Fasting glycemia (mmol/L)	8.4 [6.9 – 10.2]	8.3 [7.2 – 8.7]	0.49
HbA1c (%)	7.7 [7.6 - 8.1]	7.5 [7.4 - 7.9]	0.19
Total cholesterol (mg/dL)	143 [116 - 172]	188 [139 - 223]	0.23
HDL (mg/dL)	40.5 [35.8 - 48.3]	40 [34 - 47]	0.62
LDL (mg/dL)	71.5 [54.8 - 94.2]	77 [73 - 155]	0.28
Triglycerides (mg/dL)	151 [104 - 206.5]	183 [108 - 232]	0.39

Supplementary Table S2. Main baseline characteristics only in those patients with an unmodified antidiabetic therapy. Values refer to those patients who did not modify the antidiabetic therapy and completed all the trial. They are expressed as median [IQR] and the statistical analysis was performed through Mann-Whitney U test.
