# Supplementary material for: Combined Inositols, α-Lactalbumin, Gymnema Sylvestre and Zinc Improve the Lipid Metabolic Profile of Patients with Type 2 Diabetes Mellitus: A Randomized Clinical Trial
Source: J Clin Med. 2023 Dec 13;12(24):7650. doi: 10.3390/jcm12247650 (PMC10743679; doi:10.3390/jcm12247650)
Supplement: Supplementary file 1 [file jcm-12-07650-s001.zip › Supplementary Table S3.pdf]

Event	Supplement (n=46)	Placebo (n=28)	RR (IC 95%)	p value
Adverse events	19 (41.3%)	10 (35.7%)	1.16 (0.63-2.12)	0.32
Serious adverse events†	3 (6.5%)	4 (14.3%)	0.46 (0.11-1.89)	0.14
Fatal events	0 (0.0%)	0 (0.0%)	-	-
Adverse events with early drop out ‡	5 (10.9%)	5 (17.9%)	0.61 (0.19-1.92)	0.20
Hypoglycemia	2 (4.3%)	1 (3.6%)	2.43 (0.29-12.82)	0.43
Serious hypoglycemia§	1 (2.2%)	0 (0.0%)	-	-
Diarrhea	4 (8.7%)	1 (3.6%)	2.43 (0.29-20.70)	0.21
Nausea	2 (4.3%)	0 (0.0%)	-	-
Vomit	1 (2.2%)	2 (7.1%)	0.91 (0.16-5.13)	0.16
Abdominal pain	0 (0.0%)	1 (3.6%)	-	-
Asthenia	0 (0.0%)	1 (3.6%)	-	-
Dyspepsia	1 (2.2%)	0 (0.0%)	-	-
Hypersomnia	2 (4.3%)	0 (0.0%)	-	-
Covid-19	2 (4.3%)	0 (0.0%)	-	-
Traumatic fracture	1 (2.2%)	1 (3.6%)	0.61 (0.04-9.35)	0.36
Hypertension	1 (2.2%)	1 (3.6%)	0.61 (0.04-9.35)	0.36
Ischemic coronary artery disease (angina pectoris; acute myocardial infarction)	0 (0.0%)	2 (7.1%)	-	-
Ventricular tachycardia	0 (0.0%)	1 (3.6%)	-	-
Headache	1 (2.2%)	1 (3.6%)	0.61 (0.04-9.35)	0.36
Polyuria	0 (0.0%)	1 (3.6%)	-	-
Transitional cell carcinoma of the bladder	0 (0.0%)	1 (3.6%)	-	-

Supplementary Table S3. Overview of the adverse events occurring during the entire study. All the events were classified according to MedDRA®, version 26.0. In the analysis of the safety all the patients who were exposed to at least one dose of the the treatment were included. † In the study group, serious adverse effects include serious hypoglycemia, pneumonia due to Covid-19 and traumatic fracture; in the placebo group the serious adverse effects include recurrent angina pectoris, ventricular tachycardia and transitional cell carcinoma of the bladder. § This event occurred in only one patient who was under basal insulin therapy. ‡ The adverse effects determining an early drop out were already reported in the flow chart in Figure 1. Risks of the adverse effects were estimated by Relative Risk (RR) calculation with 95% interval of confidence. No statistically significant variations between the two groups.
